# Annual Report of the Officers of the Indiana Hospital for the Insane

**Published:** 1862-05

**Authors:** 


Annual Report of the Officers of the Indiana Hospital for the Insane. For the year ending Oct. 31, 1861. By JAMES S. ATHON, M.D., Superintendent. Indianopolis.

This report is similar in its character and interest to the two preceding, though printed on poorer paper and in inferior style. These several reports in relation to the Insane, afford much material for reflection and intesting inquiry, especially in relation to the causes of mental derangement. For instance, these three reports state the probable causes of Insanity in 3073 cases. Of these 461 are ascribed to "ill-health;" 230 to "domestic trouble;" and 177 directly to "intemperance." But it is our present purpose simply to announce the publication of these reports, without commenting on their contents.

